# The enigma of primary hypertension in childhood

**DOI:** 10.3389/fcvm.2022.1033628

**Published:** 2022-11-04

**Authors:** Bonita Falkner

**Affiliations:** Sydney Kimmel School of Medicine, Thomas Jefferson University, Philadelphia, PA, United States

**Keywords:** blood pressure, hypertension, children, adolescents, obesity

## Abstract

Beginning in the 1970s, hypertension in children and adolescents has been defined as systolic and/or diastolic blood pressure (BP) that is equal to or greater than the 95th percentile of the normal BP distribution in healthy children. The definition of hypertension in adults is based on longitudinal data that links a BP level with an increased risk for subsequent adverse outcomes related to hypertension including heart failure, kidney failure, stroke, or death. The statistical definition of hypertension continues to be used in childhood because there have been no data that link a BP level in childhood with a heightened risk for adverse outcomes in adulthood. Findings from clinical and epidemiologic research have advanced understanding of high BP in childhood. While hypertension in some children can be secondary to underlying kidney, cardiovascular, or endocrine disorder, it is now known that primary (essential) hypertension can be present in childhood. The prevalence of hypertension in childhood is approximately 2–5% and another 13–18% of children and adolescents have elevated BP and are at heightened risk for developing hypertension. The leading cause of childhood hypertension is primary hypertension, especially in adolescents. For children and adolescents with secondary hypertension, the treatment can focus on managing the underlying cause of hypertension. Less is known about managing primary hypertension in childhood, including diagnosis, evaluation, treatment, and possibilities for prevention. The phenotype of primary hypertension in childhood and recent findings will be discussed.

## Introduction

Hypertension in adults is based on longitudinal data that defines a BP level that increases the risk for subsequent adverse outcomes including heart failure, kidney failure, stroke, or death. No such data have been available for children or adolescents. In the 1970s, reference BP data became available on the normal distribution of BP levels in children and adolescents according to age and sex (1, 2), and later, height was added in the distribution (3). Based on that data, the definition of hypertension in childhood became a systolic and/or diastolic BP ≥ 95th percentile according to age, sex, and height. Therefore, unlike the outcome-based definition in adult hypertension, the pediatric definition of hypertension in childhood has remained a statistical definition.

With available tables to identify abnormal BP in children and adolescents, early pediatric guidelines on childhood hypertension focused on diagnostic testing to identify a secondary cause for the hypertension in a child or adolescent including renal, cardiovascular, or endocrine disorder; and primary (essential) hypertension was considered a disorder limited to adulthood. The childhood obesity epidemic has changed this perspective.

## The primary hypertension phenotype in childhood

An analysis of the two separate National Health and Nutrition Survey (NHANES) periods in 2004 reported a population increase in BP levels among children and adolescents that was largely related to an increase in overweight/obesity (4). A subsequent analysis of sequential NHANES periods demonstrated that independent predictors of increasing BP levels in children and adolescents were body mass index, waist circumference, and dietary sodium intake (5). Clinical reports on hypertensive adolescents noted a strong association of obesity with hypertension (6). Other cross-sectional clinical studies on adolescents with hypertension described a strong association with obesity and also noted a substantial prevalence of the left ventricular hypertrophy (LVH) in the adolescents with hypertension (7–9). Based on these clinical findings, the Fourth Report on childhood hypertension (10) recommended an echocardiogram as part of the clinical evaluation in hypertensive children to determine the presence of hypertension-associated LVH as a measure of target organ damage (TOD); screening for other risk factors including lipids and glucose was also recommended. Subsequently, dyslipidemia was also reported in children with obesity-associated hypertension (11). Therefore, it was becoming apparent that childhood hypertension characterized by obesity and cardiometabolic risk factors is a phenotype very similar to primary hypertension in adults.

## Blood pressure tracking in childhood

Identifying primary hypertension in asymptomatic healthy children and adolescents can be challenging. The BP reference tables developed from cross-sectional data on BP measurements in large samples of healthy children demonstrate a normal progressive increase in BP level from early childhood through adolescence consistent with normal childhood growth. Clinically, it known that BP measurements tend to be variable, especially in early childhood and it was not known whether a BP level at a given BP percentile consistently follows the same percentile with growth. To determine whether BP at a high BP percentile tracked from childhood through adolescence, Chen and Wang (12) performed a systematic review and meta-regression analysis that included 50 published reports of longitudinal childhood studies that included BP measurements in asymptomatic healthy children. Overall, the tracking correlation coefficient varied considerably. With further analysis, it was determined that the tracking coefficient varied according to baseline age and length of follow-up. For children <5 years at baseline measurement, the tracking coefficient was insignificant at 0.18. However, by baseline age 8–9 years, the systolic BP tracking coefficient was significant and consistent at 0.40–0.43 up to age 18 years. These findings indicate that by mid-childhood, systolic BP levels at the higher BP percentiles tend to track and indicate a heightened risk for primary hypertension.

## Blood pressure trajectories from childhood to adulthood

Analysis of longitudinal data on BP and risk factors associated with abnormal BP in prospective studies that began in childhood and extended into early adulthood provide a life-course perspective on childhood origins of primary hypertension (13–15). Theodore et al. (13) analyzed data on 975 children who were enrolled at age 7 years and followed with repeated measurements to age 38 years. At age 38 years, participants were stratified based on BP status to hypertension, pre-hypertension, high normal BP, and normotensive. Using group-based trajectory modeling on BP curves for each BP status group at age 38 years, there were four systolic BP curves identified. According to BP classification at age 38 years, there was a clear separation of the systolic BP trajectory groups by 11 years of age. Significant risk factors identified for the hypertensive and pre-hypertensive groups were low birth weight, family history of hypertension, male sex, higher body mass index (BMI), and cigarette smoking. BP trajectory analysis was also performed by Hao et al. (14) in a cohort of 546 participants enrolled in early childhood and followed prospectively to adult age 30 years. Their data included measures of the left ventricular mass index (LVMI) and carotid intimal medial thickness (cIMT) as intermediate measures of cardiovascular injury. The authors identified three separate BP trajectory curves, designated as low-increasing, moderate-increasing, and high-increasing. By age 10 years, there was a separation of the systolic BP trajectory curves. Systolic BP was above 120 mmHg by age 15 years in the high-increasing group. At age 30 years, LVMI and cIMT were highest in the high-increasing systolic BP group. These reports demonstrate that higher systolic BP levels in childhood can progress to hypertension and pre-hypertension by early adulthood with markers of cardiovascular injury.

Subsequent analysis of data in longitudinal cohorts examined associations of higher BP status in childhood with intermediate markers of cardiovascular disease (CVD) in adulthood, and Yang et al. (16) conducted a systematic review of childhood to adulthood longitudinal cohort studies. The meta-analysis determined that BP levels ≥90th percentile in childhood or adolescence were significantly associated with risk for LVH, with a pooled odds ratio (OR) 1.40 (95% CI = 1.20–1.64); vascular stiffness ascertained by pulse wave velocity (PWV) OR 1.83 (95% CI = 1.39–2.40); and high cIMT, OR 1.60 (95% CI = 1.29–2.00). This systematic review also identified some associations of abnormal BP in adolescence with clinical CVD and mortality in adulthood. More recently, investigators in the International Childhood Cardiovascular Cohorts (i3C) Consortium reported an analysis on longitudinal data in the childhood to adulthood cohorts to determine whether there was an association of risk factors in childhood with CVD events in adulthood. Childhood risk factors considered were systolic BP, BMI, total cholesterol level, triglyceride level, and youth smoking. For each risk factor, age- and sex-specific z-scores were determined and a combined-risk z-score was calculated. There were 319 fatal cardiovascular events among 38,589 participants. The hazard ratio for the combined-risk z-score was 2.71 (95% CL, 2.23 to 3.29) per unit increase. A similar finding was found in the analysis of 779 fatal and non-fatal cardiovascular events that occurred in 20,656 participants (17). The results in this publication provide evidence that high BP and other cardiovascular risk factors in childhood are associated with adverse CVD outcomes in mid-adulthood.

## Markers of cardiovascular injury in youth

The above describes associations of abnormal BP in youth with intermediate markers of cardiovascular disease, commonly termed target organ damage (TOD), in early adulthood. Moreover, LVH is frequently found in adolescents with confirmed primary hypertension, having systolic BP consistently ≥95th percentile (7–9). In adolescents with hypertension, confirmed by ambulatory blood pressure monitoring, subclinical, alterations in cognitive function have also been demonstrated. Compared to normotensive adolescents, adolescents with hypertension had scores significantly lower on tests of memory, attention, and executive function (18, 19).

Additional clinical studies also reported LVH in overweight/obese adolescents with pre-hypertension and hypertension (20, 21). These reports found that elevated BP and obesity were both independently associated with LVH in youth and led to questions on what BP level was linked with TOD in youth. The Study of Hypertension in Pediatrics, Adult Hypertension Onset in Youth (Ship Ahoy) project was designed to address this issue. Investigators sought to determine if the BP threshold for LVH in youth was below the 95th percentile and whether there were other cardiometabolic factors that raised the risk for TOD in adolescents. Healthy adolescents, aged 11–19 years, were enrolled, including participants with an average BP level >95th, and stratified into three groups according to office systolic BP measurements (average of six measurements from two separate visits): low risk = BP < 80th percentile; mid risk = 80th to <90th percentile; and high risk ≥ 90th percentile. The groups were matched by age and demographics with a slight difference in body mass index (BMI). Mean BP, LVMI, and prevalence of LVH increased across groups: For LVH, the low BP group prevalence = 13%, mid group = 21%, and high group = 27%. Systolic BP percentile was found to be a significant determinant of LVMI, and the 90th percentile for systolic BP resulted in the best balance between sensitivity and specificity in predicting LVH (LVMI > 38.8 gm/m^2^.^7^). These results demonstrate that abnormal cardiac mass can be found at BP levels below the 95th percentile in adolescents (22). Advancements in echocardiology enable the measurement of cardiac function. In adults, cardiac function changes in left ventricular strain and diastolic function are found to precede decreases in left ventricular ejection fraction and CVD events. Further analysis of echocardiographic data in the Ship Ahoy cohort examined the effect of systolic BP level, across the three BP groups, on left ventricular strain and diastolic function. The mid-risk and high-risk participants had significantly lower left ventricular ejection fraction and peak global longitudinal strain than the low-risk group. The high-risk group had greater left ventricular strain and lower diastolic function compared to the mid-risk and low-risk groups. BP and adiposity were both statistically significant determinants of impaired left ventricular systolic and diastolic function (22). These novel findings indicate that even subclinical changes in cardiac function can be detected in adolescents with primary hypertension.

As noted above, increases in PWV, a measure of vascular stiffness, are commonly associated with hypertension in adults. Analysis of PWV data in the Ship Ahoy cohort was conducted to determine whether BP-related increases in arterial stiffness were present in adolescents with elevated BP. Carotid-femoral PWV increased across the BP groups from low-risk group to the high-risk groups. Aortic distensibility and compliance were greater in the low-risk group than the mid-risk and high-risk groups. Significant determinants of arterial stiffness were sex, age, adiposity, BP, and low-density lipoprotein (LDL). PWV and aortic compliance were significantly associated with measures of TOD (systolic and diastolic cardiac function and urine albumin/creatinine ratio) after controlling for BP level. These results indicate that BP-related vascular stiffness can also be detected at BP levels below the 95th percentile (23). Moreover, in this analysis, low-density lipoprotein (LDL) level was significantly associated with vascular stiffness. The Ship Ahoy investigators conducted additional analysis to determine whether there is a metabolic phenotype associated with TOD in adolescents with elevated BP or hypertension. A cardiovascular disease risk score was developed using the number of CVD risk conditions including hypertension, dyslipidemia, obesity, and insulin resistance present in each participant. Generalized linear models indicated that dyslipidemia and insulin resistance were independently associated with markers of diastolic dysfunction, and increased systolic BP was associated with all markers of TOD (24). These publications from the Ship Ahoy project describe a high-risk phenotype in adolescence for subsequent cardiovascular disease that includes evidence of TOD as well as multiple risk factors even at BP levels below the 95th percentile.

## Prevalence and diagnosis of primary hypertension in childhood

Reports on the prevalence of primary hypertension in childhood vary based on the population studied, location of the population, how the BP is measured, the number of BP measurements, the definition of hypertension, and the reference data on normative BP levels for age and sex. The overall estimated prevalence of hypertension in childhood is approximately 2–5% and the prevalence of elevated BP is 13–18% (25, 26), with higher rates in adolescence compared to childhood. The prevalence of secondary hypertension is approximately 1% and is generally identified in early childhood or in children with markedly elevated BP (27, 28). It is now established that primary hypertension is the most common type of hypertension in the young, especially in adolescence.

Some children are at greater risk for primary hypertension including children with overweight or obesity and children with a history of low birthweight. A sub-optimal diet can be a target for intervention in childhood and adulthood. Dietary salt intake is high in childhood with most children and adolescents exceeding recommended limits in sodium intake, largely due to the consumption of processed foods (29).

In clinical practice, it is challenging to recognize abnormal BP levels in asymptomatic otherwise healthy children and adolescents. Although BP measurement in children, beginning at age 3 years, is now standard practice in primary care settings, examinations of electronic health records have demonstrated under-recognition and underdiagnosis of hypertension in children and adolescents (30, 31). Evidence-based guidelines are available to facilitate diagnosis, evaluation, and management of hypertension including both primary and secondary hypertension (27, 28, 32). However, recognizing abnormal BP levels and taking appropriate steps in follow-up continue to be difficult to achieve in primary care settings (33). Electronic medical record alerts (34) and clinical decision support systems (35) improve recognition of abnormal BP in children somewhat but documentation of diagnosis and appropriate follow-up remains sub-optimal. A necessary step in identifying and managing abnormal BP is the accurate measurement of BP. A flawed technique used in the measurement of BP in both children and adults results in inaccurate readings. Staff who measure BP in children and adolescents in a primary care setting should be trained in a standard BP measurement protocol (27, 28, 36). It is also important to use a BP monitor that is validated for accuracy in children (37, 38).

Strategies and tools are still needed to help primary care clinicians overcome the barriers to appropriate screening, recognition, and confirming abnormal BP in children and adolescents (39). This is especially important because the rates of childhood obesity, the major risk factor for pediatric primary hypertension, are increasing (40). Moreover, an analysis of data in the prospective cohort Coronary Artery Risk Development in Young Adults (CARDIA) study found that young adults, with stage 1, stage 2 hypertension, and even elevated BP, had a significantly higher risk for subsequent CVD events compared to those who had normal BP before age 40 years (41). With the increasing prevalence of hypertension in adolescents, it is expected that the prevalence in young adults will also increase. In the United States, CVD projections estimate significant increases in CVD among adults in future decades, including hypertension (42). Detection and effective management of hypertension and elevated BP in adolescence, as well as primordial prevention beginning in early childhood, would have a substantial impact on stemming the rising tide of CVD in adults. Adolescents should be able to enter adulthood with a normal BP.

## Author contributions

The author confirms being the sole contributor of this work and has approved it for publication.
